# Evaluation of the IS*6110 *PCR assay for the rapid diagnosis of tuberculous meningitis

**DOI:** 10.1186/1743-8454-4-10

**Published:** 2007-11-02

**Authors:** Poonam S Deshpande, Rajpal S Kashyap, Sonali S Ramteke, Khushboo J Nagdev, Hemant J Purohit, Girdhar M Taori, Hatim F Daginawala

**Affiliations:** 1 Biochemistry Research Laboratory, Central India Institute of Medical Sciences, Nagpur, India, 440010; 2 Environmental Genomics Unit, National Enviromental Engineering Research Institute, Nehru Marg, Nagpur, India, 440020

## Abstract

**Background:**

Tuberculous meningitis (TBM) is one of the common clinical manifestations of extra-pulmonary tuberculosis. It is difficult to diagnose due to a lack of rapid, sensitive, and specific tests. Newer methods, which are easy and reliable, are required to diagnose TBM at an early stage. Thus our aim was to evaluate the polymerase chain reaction (PCR) technique, using primers directed against the IS6110 gene, for the detection of *Mycobacterium tuberculosis *in the CSF, for the diagnosis of TBM patients.

**Methods:**

An in-house IS*6110 *PCR method using a specific pair of primers designed to amplify the insertion sequence, IS*6110*, in the *M. tuberculosis *genome was used to analyze CSF. A total of 80 CSF samples from different groups of patients were studied (confirmed TBM n = 35, clinically suspected TBM n = 16, non-TBM infectious meningitis n = 12, non infectious neurological diseases n = 17).

**Results:**

PCR gave a sensitivity of 91.4% and specificity of 75.9% for the diagnosis of TBM in patients with TBM confirmed by culture. In 16 clinically diagnosed, but unconfirmed, TBM cases PCR was positive in 10 (62.5%) cases. There were seven (24.1%) PCR-positive cases among the 29 patients with non-TBM and non-infectious neurological disease.

**Conclusion:**

We conclude that the performance of an in-house IS*6110 *PCR assay is valuable in the rapid diagnosis of tuberculous meningitis.

## Background

Tuberculosis (TB) is one of the major causes of morbidity and mortality worldwide. India has about 1.8 million new cases of TB annually, accounting for a fifth of new cases in the world – a greater number than in any other country [1]. Among, extra-pulmonary TB, tuberculous meningitis (TBM) leads to multiple central nervous system (CNS) complications and remains a major health problem in underdeveloped and developing countries [2]. Delayed treatment of TBM is associated with high mortality and with neurological problems, which underscores the importance for early diagnosis [3].

Confirming the clinical suspicion of TBM has always been problematic. Acid-fast bacilli (AFB) staining of cerebrospinal fluid (CSF) has a very low sensitivity [4]. Although conventional bacterial culture is the gold standard for diagnosis, the inherent time limitation of the culture-based test, limits its value [5,6]. The culture of *M. tuberculosis *from CSF takes 4–6 weeks and leads to a delay in diagnosis [7,8]. Analysis of CSF using antibody detection is suggestive but not diagnostic of TBM [9]. In the absence of any reliable diagnostic methods, various immunological and molecular methods have been advocated including ELISA [10] for demonstration of *M. tuberculosis *antigen and antibodies, T cell based assay for IFN gamma estimation (ELI SPOT), adenosine deaminase assay [11,12], and polymerase chain reaction (PCR) [13,14]. However, all the above-mentioned methods are still being evaluated.

Rapid techniques based on nucleic acid amplification such asPCR have been reported to be more sensitive and specific as they attempt to detect specific DNA sequences from the organism under investigation. Several *M. tuberculosis *specific DNA sequences have been evaluated in different laboratories including MBP-64, 65 kDa antigen and IS*6110 *[14]. The reliability of PCR depends on the amplification of DNA with primers specific for different target sequences in the mycobacterial genome, and on optimal DNA isolation and PCR procedures [15]. The observed sensitivity and specificity of the PCR for *M. tuberculosis *in clinical samples differs greatly among the different laboratories ranging from 50–90% and 60–100%, respectively [6]. The repetitive nature of IS6110 insertion sequence in *M. tuberculosis *genome makes it an attractive target for PCR amplification, as it could contribute to a higher degree of sensitivity of the assay [16,17]. Several studies have been undertaken to evaluate the efficacy of IS*6110 *sequence for the diagnosis of tuberculosis [5,8,14]. In our study, we describe our experience with the IS*6110 *based PCR assay to detect *M. tuberculosis *DNA in CSF samples of TBM and non-TBM cases in our Institute.

## Methods

CSF samples from a total of 80 patients were analysed. These consisted of confirmed and clinically suspected TBM patients, n = 51, patients with other infections (pyogenic meningitis, n = 5, viral meningitis, n = 7), and control subjects with non-infectious neurological disorders, n = 17. Patients for this study were admitted to the Neurology Department of Central India Institute of Medical sciences (CIIMS), Nagpur between September 2005 and December 2006. All patients were above the age of 20 years and had given written consent for the study. CSF samples for ADA estimations and other tests were obtained before starting any specific treatment in all cases of neurological disorders including viral, bacterial, and mycobacterial meningitis. The Institutional Ethics Committee of Central India Institute of Medical Sciences, Nagpur, approved the study. To establish a diagnosis of meningitis, 2–5 ml of CSF was withdrawn from patients using a lumbar puncture. CSF was then subjected to routine biochemical and pathological analysis including Gram, India ink, and AFB staining and culturing. Diagnosis of TBM and non-TBM was based on criteria described below.

### Patient groups

#### 1. *Tuberculous meningitis *patients (n = 51)

A: Clinically confirmed cases (n = 35): Confirmed by the presence of *M. tuberculosis *in CSF by staining and/or culture.

B: Clinically suspected patients (n = 16): This group had negative cultures with all of the following observations:

a: Sub-acute or chronic fever with features of meningeal irritation such as headache, neck stiffness and vomiting, with or without other features of CNS involvement.

b: CSF samples showing raised protein levels, and/or decreased glucose (CSF: blood glucose ratio < 0.5), and/or pleocytosis with lymphocytic predominance.

c: Good clinical response to anti-tuberculous drugs.

#### 2. Non-TBM patients

##### A) Non-TBM infectious meningitis (n = 12)

A: Pyogenic meningitis (n = 5):

Confirmed cases (n = 2): Presence of pathogenic bacteria in CSF by staining and/or culture.

Clinically suspected (n = 3): This group included the culture negative cases with all of the following observations:

a: Fever and/or signs of meningeal irritation (patients who have undergone cranial surgery to treat tumor(s), stroke, or head injury and who have received antibiotics), or high fever and/or signs of meningeal irritation with or without CNS manifestations (patients who received broad-spectrum antibiotics).

b: CSF findings showing increased proteins, decreased glucose (CSF: blood glucose ratio < 0.2), and/or pleocytosis with a predominance of polymorphonuclear cells.

c: Good clinical response to road-spectrum antibiotics.

B: Viral meningitis patients (n = 7): This group included suspected patients with the following observations:

a: Acute onset of fever and symptoms and signs of meningeal irritation.

b: CSF samples showing mild increase in protein, glucose levels often normal, and pleocytosis, predominantly lymphocytic.

c: No clinical evidence for extra cranial tuberculosis.

##### B) Non-infectious neurological disorders group (n = 17)

All other patients who had no evidence of CNS or extra CNS bacterial or viral infections were grouped in the non-infectious/control group. Patients included in this group had chronic headache and hypertension n = 10, head injury n = 2, paraparesis, dementia, myelopathy, acute cerebellitis, and epilepsy, n = 1 each.

### Culture procedure

CSF samples (0.5 ml) were inoculated into 5 ml BioFM liquid media (Biorad, Marnes-la-Coquette, France) and incubated at 37°C. All culture samples were examined twice a week for 6 weeks. The positivity of culture was defined by the growth of mycobacteria in the liquid media.

### DNA extraction

DNA was extracted according to the CTAB-phenol chloroform extraction method. Briefly, 0.2 ml of CSF was centrifuged at 10,000 rpm for 10 min. The supernatant was discarded and the pellet suspended in 567 μl of TE buffer (Tris EDTA, pH 7.4), 30 μl 10% SDS and 3 μl proteinase K (20 mg/ml), mixed and incubated at 37°C for 1 h. After incubation, 100 μl of 5 M NaCl and 80 μl of high-salt CTAB buffer (containing 4 M NaCl, 1.8% CTAB (cetyl-trimethyl-ammonium bromide) was added and mixed followed by incubation at 65°C for 10 min. An approximate equal volume (0.7–0.8 μl) of chloroform-isoamyl alcohol (24:1) was added, mixed thoroughly and centrifuged for 4–5 min in a microcentrifuge at 12,000 rpm. The aqueous viscous supernatant was carefully decanted and transferred to a new tube. An equal volume of phenol: chloroform-isoamyl alcohol (1:1) was added followed by a 5 min spin at 12,000 rpm. The supernatant was separated and then mixed with 0.6 volume of isopropanol to get a precipitate. The precipitated nucleic acids were washed with 75% ethanol, dried and re-suspended in 100 μl of TE buffer.

### Polymerase chain reaction (PCR)

In each independent PCR assay, test results were compared with the results for one positive and one negative control. The positive controls included the DNA of H_37_Rv strain provided by Colorado State University, Fort Collins, USA, (Contract No 1-A1-40091). Negative control included PCR grade water.

Identification of *M. tuberculosis *was done using a specific pair of primers designed to amplify an insertion sequence IS*6110 *in the *M. tuberculosis *complex and the expected band size was about123-bp. The sequence of these primers, T4 and T5, are: 5'-CCT GCG AGC GTA GGC GTC GG 3' and 5' CTC GTC CAG CGC CGC TTC GG 3' respectively. A 50 μl reaction contained 10× assay buffer (Bangalore Genei, Bangalore, India), 10 mM dNTP's (Bangalore Genei), 10 pmole of each primer (SIGMA-GENOSYS, USA), 2.5 units Taq DNA Polymerase (Bangalore Genei) and 5 μl of extracted DNA. Amplification was carried out in a thermal minicycler (peqlab Biotechnologie GmbH, Erlangen, Germany), which involved 40 cycles of denaturation at 94°C for 2 min, annealing of primers at 68°C for 2 min, and primer extension at 72°C for 1 min. The amplification products were separated on 2% agarose gels, visualized on a UV- light transilluminator (Biotech R & D Laboratories, Yercud, Salem, India) and photographed.

## Results

**Figure 1:**
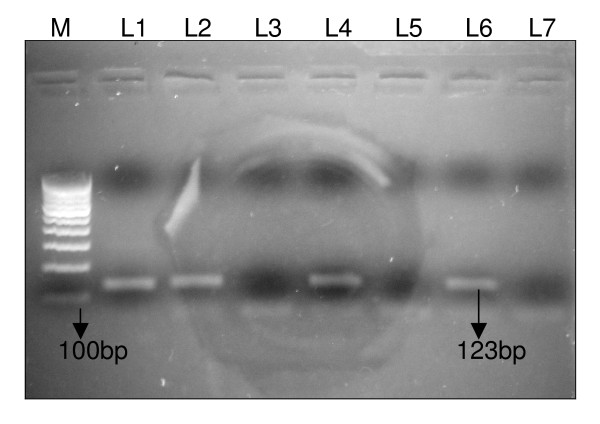
Amplification of the 123 bp product of *M. tuberculosis *by PCR. PCR products were analyzed by electrophoresis on 2% agarose gel. M represents 100 bp DNA ladder. L1: positive control DNA (*M. tuberculosis *strain H_37_Rv). L2, L4, L6: clinically positive TBM samples. L3, L5: non-TBM samples. L7: negative control.

Figure 1 shows the electrophoresis product of the 123 bp amplification of IS*6110 *sequence of *M. tuberculosis *by PCR. M represents 100 bp DNA ladder. Lane 1 (L1) represents the positive control DNA (*M. tuberculosis *strain H_37_Rv). L2, L4, L6 show clinically positive TBM samples, and L3 and L5 represent non-TBM samples. L7 is a negative control.

The results of the IS*6110 *PCR assay in the TBM and non-TBM CSF samples are shown in Table 1. The IS*6110 *PCR assay was positive in 91.4% (32/35) of confirmed TBM cases. However, in the clinically diagnosed TBM patients, but not confirmed by culture, the sensitivity of IS*6110 *PCR assay was 62.5% (10/16) (Table 1). Using culture as the gold standard, the PCR assay showed a sensitivity of 91.4% and specificity of 75.9% with a positive predictive value (PPV) = 85.7% and negative predictive value (NPV) = 71.9%). The overall sensitivity and specificity (including the TBM confirmed and clinically suspected cases together) of PCR was thus found to be 82.4% (NPV = 71.9%) and 75.9% (PPV = 85.7%) respectively.

**Table 1:** Numbers and percentages of patients showing positive or negative PCR in CSF samples.

**Category**	**PCR +ve**	**PCR -ve**
A] TBM (51)	42 (82.4%)	
(i) Confirmed TBM * (35)	32 (91.4%)	3
(ii) Clinically diagnosed TBM ** (16)	10 (62.5%)	6

B] Non TBM (29)	7 (24.1%)	
(i) Infectious ^# ^(12)	3 (25.0%)	9
(ii) Non-infectious^## ^(17)	4 (23.5%)	13

*Confirmed TBM = culture +ve/smear -ve

**Clinical diagnosed TBM = culture -ve/smear -ve

^#^Pyogenic meningitis (5), viral meningitis (7)

^## ^Chronic headache & hypertension (10), head injury (3), paraparesis, dementia, myelopathy, acute cerebellitis (1 each)

In the non-TBM infectious control group, PCR was positive in 3 samples (2 viral meningitis and 1 pyogenic meningitis). In non-infectious control group PCR was found to be positive in 4 samples (2 chronic headache, and 1 each of epilepsy and acute inflammatory demyelinating myelitis, Table 1).

We have also compared our PCR data with the culture results, which showed a sensitivity of 68.6% (NPV = 79.3%) and a specificity of 79.3% (PPV = 85.3%) as compared to PCR showing 82.4% sensitivity (NPV = 71%) and 75.9% specificity (PPV = 85.7%), (Table 2).

**Table 2:** Sensitivity and specificity of PCR test compared to CSF culture.

**Test Result**	**Final Diagnosis**		**Sensitivity**	**Specificity**	**PPV**	**NPV**
**PCR**	**TBM **(N = 51)	**Non TBM **(N = 29)				
					
Positive	42	07	82.4%	75.9%	85.7%	71%
Negative	09	22				

**Culture**						
Positive	35	06	68.6%	79.3%	85.3%	79.3%
Negative	16	23				

## Discussion

TBM is one of the common clinical manifestations of extra-pulmonary tuberculosis. There has been a rising trend of TBM in developing countries like India in the past two decades. The detection of TBM is difficult to establish because of its pleomorphic clinical presentation and variable CSF cellular content and biochemical parameters, similar to that of partially treated pyogenic meningitis cases. Delayed diagnosis and treatment may be associated with many serious CNS complications [18]. Hence, rapid detection of *M. tuberculosis *is of vital importance for the proper diagnosis and management of tuberculous meningitis. Polymerase chain reaction is considered to be one of the most specific diagnostic methods amid the many rapid methods studied. PCR is the method of choice for the diagnosis of tuberculosis in cases where the suspicion is high but AFB staining is negative [19]. Many investigators have described the rapid detection of *M. tuberculosis *by PCR, and have reported a high degree of sensitivity using this method [20-24]. Although, IS6110 PCR is not a novel diagnostic tool, it is believed that more studies are required to establish its utility in the diagnosis of TBM [20]. This study was planned to evaluate the efficacy of an in-house IS*6110 *PCR assay to detect *M. tuberculosis *DNA in CSF samples collected in our Institute.

The characteristic of the IS*6110 *PCR assay is that it should be capable of detecting the presence of *M. tuberculosis *DNA in all the samples proven to be TBM positive using culture as the gold standard [20]. The present study was performed blinded to evaluate the IS*6110 *PCR assay for detection of *M. tuberculosis *in CSF samples available from our hospital, using an in-house protocol. In our study, the IS*6110 *PCR assay detected the presence of *M. tuberculosis *DNA in 91.4% (32/35) cases of confirmed TBM and in 62.5% (10/16) cases of clinically diagnosed TBM which were negative for mycobacterial culture, but had a clinical index of suspicion for TBM.

Our results show agreement with some previous studies, which showed 85–98% sensitivity [20,25,26] and a disparity to various other studies, which had a low sensitivity of 32–75% [27-30]. Rafi *etal *used a DNA extraction protocol involving CTAB detergent, which enhances PCR positivity in CSF samples [20]. We have also used the same detergent along with the phenol-chloroform method for DNA extraction. The reason for lower sensitivity in many studies is unclear. However, it could be due to the low volume of CSF available, inefficient lysis of cells and/or loss of DNA during purification or different methods used for extraction of DNA [6]. In our study, the reason for PCR negativity in six out of 16 clinically diagnosed TBM cases could be the presence of a low number of bacteria or poor lysis of bacteria, or possibly the presence of some PCR inhibitors like bacterial contaminants, phenol etc. in the CSF samples. Sometimes the tough cell wall of *M. tuberculosis *makes the isolation of target DNA difficult [30]. There have been a few reports citing the existence of mycobacterial strains that lack IS*6110 *element and therefore, infection with an IS*6110 *negative strain cannot be ruled out [30-33]. Other sequences such as Ag85, MPB-64, 65 kDa, and 38 kDa have also been used as target for PCR amplification. But IS*6110 *PCR has shown enhanced sensitivity and specificity as a single step PCR assay [14,34]. Nested PCR assay for diagnosis of TBM has provided a considerable increase in sensitivity and specificity of DNA amplification compared to conventional single-step PCR assay [35,36]. However, the nested PCR assay using CSF samples has yet to be widely used in TBM diagnosis, due to its laborious and time-consuming procedure, which carries a high risk of sample contamination [36,37].

For the detection of overall TBM cases, the IS*6110 *PCR assay was useful in terms of sensitivity (82.4%) and specificity (75.9%) as compared to culture results showing 68.6% sensitivity and 79.3% specificity in overall TBM and non-TBM cases (Table 2). Therefore, it has been found that IS*6110 *PCR is a good supportive method for rapid diagnosis of clinically diagnosed TBM, particularly where AFB staining and cultures are negative. Some other studies have also suggested that CSF PCR for *M. tuberculosis *is more sensitive than AFB staining and culture in cases of clinically suspected TBM that responded to empirical treatment [38].

Although the PCR can amplify the specific DNA sequence by thousands of times within a few hours, the exquisite specificity of PCR is also its main potential drawback. Out of 29 non-TBM cases, PCR was positive in 3 cases of infectious group (1 for pyogenic meningitis and 2 for viral meningitis). In our study, the two PCR positive cases of viral meningitis also had a positive TB culture, which suggests they could be classified as case of mixed meningitis. Mixed bacterial meningitis involving mycobacteria and other bacteria have also been described earlier [39].

PCR was also positive in four cases of non-infectious non-TBM group, which were actually diagnosed for chronic headache (two cases), epilepsy and myelopathy myelitis (one each). One reason for such false positive results could be cross contamination with the amplified DNA product in the laboratory. This is a well-recognized problem in other laboratories [40]. In spite of a small number of CSF samples showing a positive result in the control groups, the PCR assay was allied with a specificity of 75.9%.

Despite the improved sensitivity and specificity of PCR technique, AFB and culture remain an important technique for diagnosing TBM. In this study, if the PCR method had been accepted as the only diagnostic criteria, it would have missed three culture confirmed cases.

In a meta-analysis of in-house and commercial protocols used in the diagnosis of TBM, the results revealed that commercial tests had an increased specificity but low sensitivity (56%). This was in stark comparison to the substantial variability of the test results seen whenever an in-house protocol was used [41]. Also the high cost involved for commercial assays make it a significant drawback, especially in developing countries like India. In such settings, the role of an in-house assay turns out to be useful in aiding the clinician towards a diagnosis of TBM, with a reliable degree of specificity and sensitivity.

## Conclusion

Our study made it evident that in-house IS*6110 *PCR is a rapid and cost-effective diagnostic test for TBM that shows good sensitivity and specificity. This can be adopted as a method of choice for the diagnosis of *mycobacterial *infections in cases where suspicion is high, in combination with other clinical criteria.

## Competing interests

The author(s) declare that they have no competing interests.

## Authors' contributions

PSD carried out the study design, data collection, data interpretation, literature search, and manuscript preparation; RSK and HJP participated in the preparation of the manuscript, data interpretation, and study design; SSR and KJN contributed in collection of samples and data interpretation, GMT provided assistance in preparation of the manuscript, data interpretation, study design, and funds collection; and HFD supervised the study design, data interpretation and manuscript preparation. All authors have read and approved the final version of the manuscript.
